# Secondary systemic lupus erythematosus after thymoma resection misdiagnosed as medically unexplained dyspnoea: A case report

**DOI:** 10.1097/MD.0000000000008944

**Published:** 2017-12-01

**Authors:** Hao Liao, Wendi Pei, Junfang Liu, Kai Wang, Tingting Xu, Xin Chen

**Affiliations:** Department of Respiratory Medicine, Zhujiang Hospital, Southern Medical University, Guangzhou, Guangdong Province, P. R. China.

**Keywords:** medically unexplained dyspnoea, systemic lupus erythematosus, thymoma resection

## Abstract

**Rationale:**

Secondary systemic lupus erythematosus (SLE) is an exceedingly rare complication of thymoma resection and is difficult to diagnose because of the insidious and nonspecific clinical manifestations. A case of SLE that occurs secondary to thymoma resection is described in this report.

**Patient concerns:**

A 43-year-old male came to our hospital with the sole symptom of dyspnea after thymoma resection initially. However, other atypical lesions of SLE occurred over time.

**Diagnoses:**

Antinuclear antibody spectrum test showed positive results and the diagnosis of SLE was obtained.

**Interventions:**

Initially the patient was treated for medically unexplained dyspnea (MUD) without much improvement. Following the diagnosis, the methylprednisolone pulse therapy and therapies of immunoglobulin and cyclophosphamide were adopted for the treatment.

**Outcomes:**

Finally, the patient's symptoms faded rapidly and favorable prognosis has been maintained till now.

**Lessons:**

This case highlights the importance of a serious and comprehensive analysis before we give the diagnosis of MUD. Additionally, ignorance of secondary SLE after thymoma resection should be prevented to avoid a delayed diagnosis and treatment.

## 1 Introduction

Thymoma is a rare tumor originating from the epithelial cells of the thymus, but it is most commonly found in mediastinum of adults. There are many pathogeneses for subsequent dyspnea after thymoma resection, including autoimmune diseases and phrenic nerve injury by the surgery.

Medically unexplained dyspnea (MUD) refers to a condition characterized by prominent dyspnea in patients without explanations of organic pathologic changes through systematic checks. When patients suffer from undetermined dyspnea, a systematic inspection on the etiological factors should be required so as to later confirm the diagnosis of MUD in a prudent and strict manner as there is a high frequency of postoperative complicated immune-related diseases, such as myasthenia gravis and systemic lupus erythematosus (SLE).^[1]^ The concurrence of autoimmune diseases can increase the difficulty of diagnosis and treatment after thymoma resection, and more importantly, if the therapy was not applied timely, the survival time for patients would be shortened. Therefore, we report a rare case of secondary SLE after thymoma resection, which only showed the single symptom of dyspnea initially.

## 2 Case presentation

**Figure 1:**
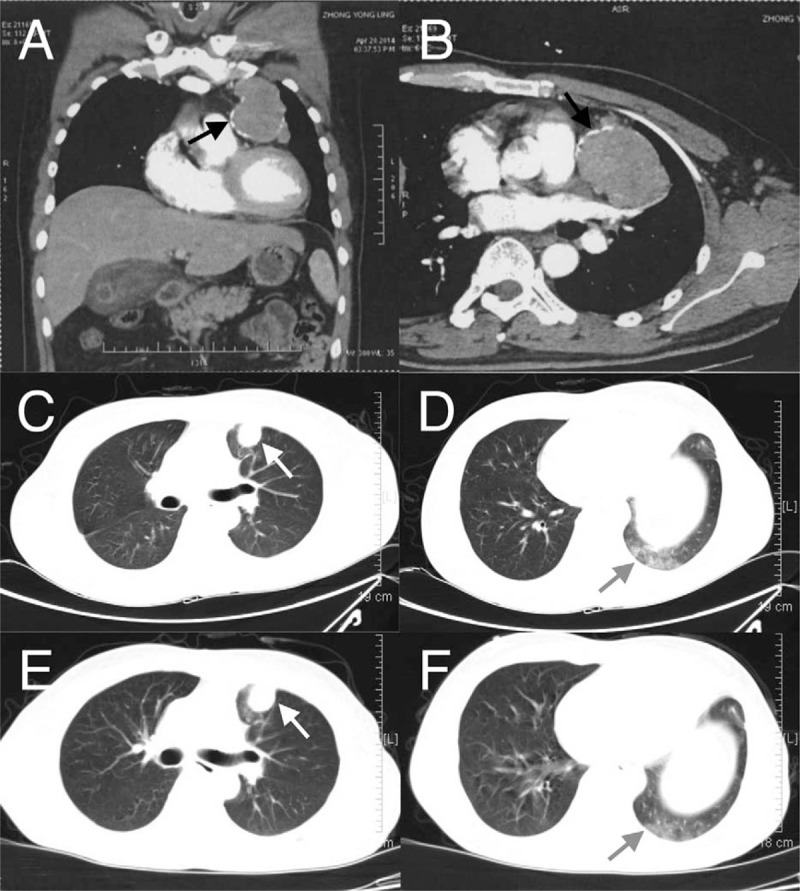
Chest CT findings. A well-defined soft-tissue mass of 10 × 8 × 6 cm was detected in the anterosuperior mediastinum and the contrast-enhanced scanning showed slight heterogeneous enhancement (A and B: black arrows). A round well-defined cystic foci in the left subpleural area (C: white arrow). A few patchy opacities in anterior segment of upper left lobe and in lower lobe (D: gray arrow). The cystic foci in the left subpleural area remained unchanged (E: white arrow). The patchy opacities dissipated slightly and the range decreased as compared with D (F: gray arrow).

**Figure 2:**
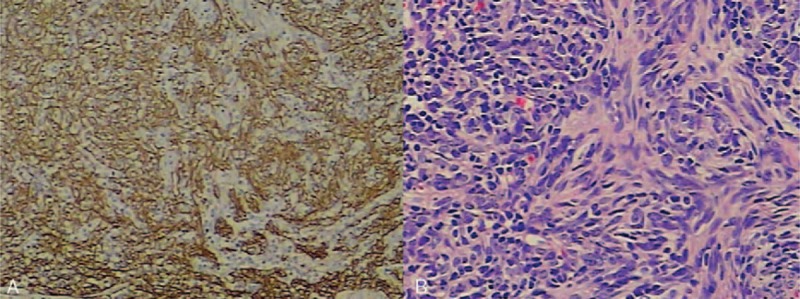
Pathological fingdings. Immunohistochemical staining (A: cytokeratin ×200) and hematoxylin and eosin staining (B: ×200) of the thymus. CK-positive thymoma cells have infiltrated into the thymus (A). Shuttle or oval epithelial cells (lack of nuclear atypia) and tumor lymphocytes are visible (B).

A 43-year-old male patient presented to the local hospital with chest distress, cough, and discomfort in anterior chest in May 2014. The pulmonary function test (PF test) suggested severe restrictive ventilatory dysfunction (48% of predicted forced expiratory volume in one second [FEV_1_]) and normal diffusion capacity (87% of predicted diffusing capacity for carbon monoxide [DLCO]), and the arterial partial pressure of oxygen (PaO_2_) was normal. Chest computed tomography (CT) examination detected a well-defined soft-tissue mass of 10 × 8 × 6 cm in anterosuperior mediastinum, and the contrast-enhanced scanning showed slight heterogeneous enhancement (Fig. 1A and B). On the basis of above results, the diagnosis of thymoma was confirmed and the excision of thymoma was performed. The final pathological finding matched the diagnosis and was interpreted as type AB thymoma (WHO classification) (Fig. 2). In the postoperative period, the patient recovered well, and the PF test suggested moderate restrictive ventilatory dysfunction (67% of predicted FEV_1_) and normal diffusion capacity (91% of predicted DLCO) 2 weeks after the surgery.

Six months after the thymoma resection, the patient gradually developed dyspnea. Postactivity inhalation accompanied by limb weakness was found upon admission to hospital, and the modified Medical Research Council (mMRC) scoring was 3. Before referral to our hospital, the patient had visited the Department of Pneumology and Department of Neurology in a large-scale local hospital and taken physical examinations and laboratory tests, although no positive results had been found. Therefore, “anxiety state” was diagnosed by the large-scale local hospital. The oxygen inhalation therapy of 2 to 3 L/min and antianxiety treatment were performed. However, the patient's symptoms had not been relieved through the therapy.

On December 26, 2014, the patient was transferred to our hospital for further treatment. In the initial treatment process, he showed no signs for improvements but great anxiety. To find out the reasons, a complete medical examination was performed and the results were as follows. The physical examinations showed that heart rate was 112 beats/min and oxyhemoglobin saturation was 94%. The blood routine showed: RBC count 3.52�T/L, hemoglobin 113 g/L, WBC count 3.89�G/L, percentage of neutrophilic granulocyte 73.6%, and no other abnormalities. The PF test suggested moderate to severe restrictive ventilatory dysfunction (53% of predicted FEV_1_) and moderately reduced diffusion capacity (56% of predicted DLCO). And no obvious abnormalities were found in ultrasonic cardiogram, urinary system, and abdominal ultrasonography. Additionally, Chest CT reexamination was performed and the results revealed well-defined cystic foci in the surgical site of thymoma resection and in the left subpleural area, a few patchy opacities in anterior segment of upper left lobe and in lower lobe and a small amount of pericardial effusion (Fig. 1C and 1D).

Furthermore, the phrenic nerve conduction time measurement for bilateral sides was performed to exclude the influence of the surgery and the results were 5.62 and 5.48 ms, respectively. The maximal inspiratory pressure and the maximal expiratory pressure were 90 and 112 cmH_2_0, respectively. These results of examination suggested normal phrenic nerve conduction function and diaphragmatic contractile function.

According to the symptom of limbs weakness, myasthenia gravis was then suspected as the common causes of dyspnoea had been excluded and myasthenia gravis is often complicated with thymoma. Thus, the Neostigmine test and fatigue test were ordered, but the negative results ruled out the diagnosis of myasthenia gravis.

As no typical symptoms and organic lesions were found in the patient, MUD was suspected greatly. However, the symptoms of the patient did not conform to the clinical diagnostic criteria of MUD proposed by American Thoracic Society (ATS), which diagnose MUD based on typical symptoms and major manifestations partly or completely induced by hyperventilation provocation test under the premise of leaving organic diseases out of account. The pulmonary fibrosis in CT scan, moderately restrictive ventilation dysfunction (53% of predicted FEV_1_), and moderately reduced diffusion capacity (56% of predicted DLCO) still cannot be explained and consequently, the diagnosis of MUD was excluded.

On the fifth day of his hospitalization, the patient complained oral ulcer and photophobia, and the chest CT examination showed a small amount of pericardial effusion. Considering that interstitial lung disease is usually associated with connective tissue diseases, which could result in the above lesions revealed by CT images, some connective tissue diseases such as systemic sclerosis, Sjögren syndrome, and SLE were taken into account. Therefore, the anti-nuclear antibody (ANA) spectrum test was performed and it showed: ANA-JZ positive 4+ (fluorescence titers >1: 999), ANA-cytoplasmic granules positive 2+ (fluorescence titers >1: 100∼1: 319), anti-nucleosome antibody 1+, antimitochondrial anybody type M2 2+, anticardiolipin antibody (−). Taken together, the diagnosis of SLE after thymectomy was further confirmed.

At last, the patient was diagnosed as having secondary SLE after thymoma resection. According to Systemic Lupus International Collaborating Clinics guideline,^[2]^ methylprednisolone pulse therapy and therapies of immunoglobulin and cyclophosphamide were adopted for the treatment in the patient. The patient's treatment course was uneventful and 14 days later, the PF test suggested normal pulmonary ventilation function (82% of predicted FEV_1_), normal diffusion capacity (85% of predicted DLCO), and normal PaO_2_. In addition, the mMRC score was 1 and chest CT suggested gradual improvements (Fig. 1E and 1F). Later, the patient was discharged when the symptoms disappeared, and 10 mg/day of methylprednisolone was recommended as maintenance therapy after discharge. Through follow-up visits, the favorable prognosis has been maintained till now.

The patient signed an informed consent document allowing his clinical history and photographs to be published, and timeline of diagnostic and therapeutic procedures is summarized in Table 1.

**Table 1:**
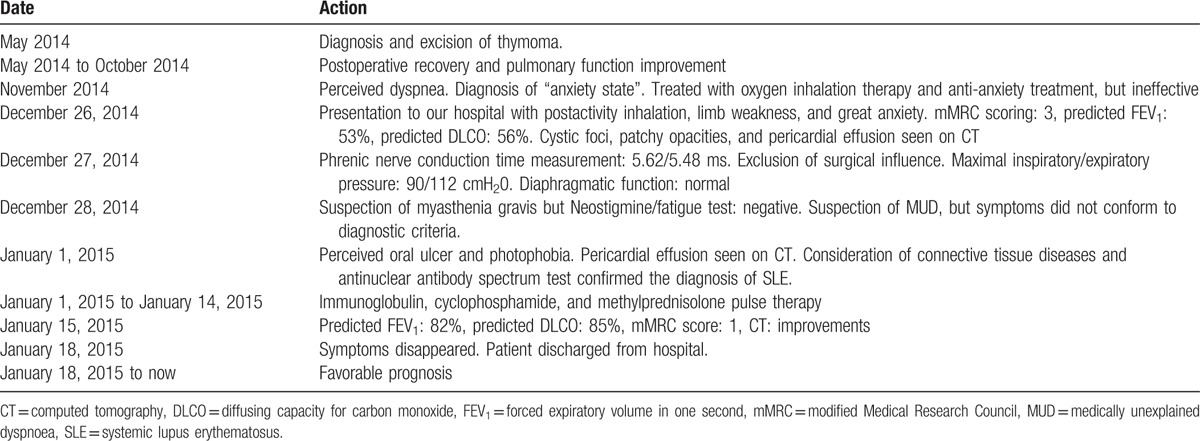
Timeline.

## 3 Discussion

In cases of SLE, common presenting symptoms include erythema, rash, arthralgia, among others. However, in this particular case of SLE after thymoma resection, only dyspnea was initially presented, followed by the appearance of great anxiety. As no typical symptoms or organic lesions had been found to explain dyspnea in this patient, MUD was suspected and the treatment was not effective until the diagnosis of SLE being made. The prompt transition in diagnosis and treatment relieved the patient's symptoms timely.

The mechanism of thymoma complicated with SLE has still not been clearly determined, whereas some scholars believe that thymosin might play a significant role in it. The action of thymosin on Th and cytotoxic T cell helps regulate and control collective immune responses. Whereas, the lack of thymosin in patients after thymoma resection leads to the reduction of the number of regulative T cells so as to further activate the autoimmune response, giving rise to SLE finally.^[3,4]^ Literatures have reported that the mortality rate can reach as high as 10% to 25% in 10 years ever since the diagnosis of SLE, usually involving in kidney, cardiovascular system, nervous system, gastrointestinal system, and respiratory system, and causing severe multisystem injuries.^[5]^

The pathogenesis of SLE has not yet been fully understood, and quite a number of scholars consider it as an autoimmunity-mediated diffuse connective tissue disease with mutual participation of T cells and B cells. Although thymoma complicated with SLE is extremely rare, the occurrence rate of SLE in patients with thymoma is 1.5% to 2.0%,^[6]^ which is much higher than that of 0.0074% to 0.159% in general healthy groups, demonstrating that there is no such occasional chance of patients with thymoma accompanied by SLE. As for the moderate restrictive ventilatory dysfunction (53% of predicted FEV_1_) and moderately reduced diffusion capacity (56% of predicted DLCO) in the PF test of this patient, we consider that because of the short disease duration of pulmonary lesions, its radiologic characteristics were not evident enough. However, the immune complexes, which deposited in pulmonary interstitium, engendered the restrictive ventilation dysfunction and reduced diffusion function, by causing damages to the capillaries of alveolar wall and then leading to the loss of functional capillaries and the formation of pulmonary interstitial fibrosis in this patient.

## 4 Conclusion

This case has reminded us that: the diagnosis of “non-organic dyspnea” should be concluded in strict accordance with the MUD diagnostic criteria proposed by ATS, and prudent diagnosis and treatment are required; when medically unexplained dyspnea happens after thymoma resection, systematic inspections should be needed to screen out all the possibilities, and after the exclusion of diaphragmatic injury and myasthenia gravis caused by excision of thymoma, other autoimmune diseases are supposed to be taken into account, particularly SLE; as in the early stage of thymoma complicated with SLE, dyspnea might be the only clinical manifestation.

## References

[1] Bernard C Frih H Pasquet F Thymoma associated with autoimmune diseases: 85 cases and literature review. Autoimmun Rev 2016;15:82–92. 26408958 10.1016/j.autrev.2015.09.005

[2] Petri M Orbai AM Alarcón GS Derivation and validation of Systemic Lupus International Collaborating Clinics Classification Criteria for systemic lupus erythematosus. Arthritis Rheum 2012;64:2677–86. 22553077 10.1002/art.34473 PMC3409311

[3] Hadaschik EN Wei X Leiss H Regulatory T cell-deficient scurfy mice develop systemic autoimmune features resembling lupus-like disease. Arthritis Res Ther 2015;17:35. 25890083 10.1186/s13075-015-0538-0 PMC4391674

[4] Omar HA Alzahrani MA Al Bshabshe AA Systemic lupus erythematosus after thymectomy for myasthenia gravis: a case report and review of the literature. Clin Exp Nephrol 2010;14:272–6. 20054604 10.1007/s10157-009-0256-5

[5] Ward MM Pyun E Studenski S Mortality risks associated with specific clinical manifestations of systemic lupus erythematosus. Arch Intern Med 1996;156:1337–44. 8651844

[6] Mevorach D Perrot S Buchanan NM Appearance of systemic lupus erythematosus after thymectomy: four case reports and review of the literature. Lupus 1995;4:33–7. 7767337 10.1177/096120339500400108

